# Fabrication of Ginsenoside-Based Nanodrugs for Enhanced Antitumor Efficacy on Triple-Negative Breast Cancer

**DOI:** 10.3389/fbioe.2022.945472

**Published:** 2022-08-12

**Authors:** Shuting Zuo, Jing Wang, Xianquan An, Zhenyu Wang, Xiao Zheng, Yan Zhang

**Affiliations:** ^1^ Department of Breast Surgery, The Second Hospital of Jilin University, Changchun, China; ^2^ Department of Anesthesiology, The Second Hospital of Jilin University, Changchun, China; ^3^ School of Biomedical Sciences and Engineering, South China University of Technology, Guangzhou, China

**Keywords:** triple-negative breast cancer, self-assembly, nanodrug, ginsenoside, biomaterial

## Abstract

There is an urgent need to identify chemotherapeutic agents with improved efficacy and safety against triple-negative breast cancer (TNBC). Ginsenosides can reportedly induce tumor cell death, invasion, and metastasis; however, poor water solubility, low oral absorption rate, and rapid blood clearance limit their clinical application. Utilizing the amphiphilic property of ginsenosides as building blocks of biomaterials, we fabricated a carrier-free nanodrug composed of ginsenosides Rg3 and Rb1 using a nano-reprecipitation method without any additional carriers. After characterizing and demonstrating their uniform morphology and pH-sensitive drug release properties, we observed that Rg3-Rb1 nanoparticles (NPs) exhibited stronger antitumor and anti-invasive effects on TNBCs *in vitro* than those mediated by free ginsenosides. Consequently, Rg3-Rb1 NPs afforded superior inhibition of tumor growth and reduction of pulmonary metastasis than the Rg3 and Rb1 mixture, with no obvious systematic toxicity *in vivo*. Collectively, our results provide a proof-of-concept that self-assembled engineered ginsenoside nanodrugs may be efficient and safe for TNBC therapy.

## Introduction

Triple-negative breast cancer (TNBC) is one of the most malignant tumors, exhibiting highly invasive characteristics (Cleator et al., 2007; Foulkes et al., 2010). Despite advances in chemotherapeutic, hormone-based, and combination drug therapies, the management of aggressive breast cancer, particularly TNBC, remains a formidable challenge (Podo et al., 2010; Garrido-Castro et al., 2019). As TNBC is more aggressive with a poorer prognosis and stronger metastatic rates than breast cancer, there are currently no approved targeted therapies (Collignon et al., 2015; Collignon et al., 2016). Chemotherapy, including anthracycline-, platinum-, and/or taxane-based neoadjuvant chemotherapy, remains the mainstay treatment for TNBC (Lebert et al., 2018; Nedeljkovic and Damjanovic, 2019; Jain et al., 2020). However, patients exhibit a poor response and inevitable adverse effects due to multiple drug resistance, along with chemotherapy-exacerbated tumor recurrence and metastasis (Bianchini et al., 2016). Therefore, there is an urgent need to identify chemotherapeutic agents that can demonstrate improved efficacy and safety against TNBC.

Ginsenosides are a series of bioactive compounds extracted from Panax ginseng, a Chinese translational herb in Asia that is well known for its beneficial pharmacological properties, including antitumor, antioxidant, and anti-inflammatory properties (Leung and Wong, 2010; Kim et al., 2015; Zheng M et al., 2018). The administration of ginsenosides has been shown to prevent tumor occurrence and progression and ameliorate cancer-related side effects (Jin et al., 2020). For example, ginsenoside Rg3, the main component of “Shenyi capsule,” was launched as an antitumor drug by the State Food and Drug Administration of China in 2000 (Chen et al., 2014; Pan et al., 2019). Rg3 has been synergistically employed with standard chemotherapeutic agents, reportedly reducing toxicity in normal tissues (Li et al., 2017). Accumulating evidence has demonstrated that combining ginsenosides with adriamycin, cisplatin, and vincristine could reverse multidrug resistance and improve antitumor effects in several cancers (Li et al., 2014; Wang et al., 2018). In addition, ginsenosides can promote antitumor immunity by regulating signal transducers, indicating their potential for efficient and safe cancer chemotherapy (Son et al., 2016; Jiang et al., 2017).

Although advances in ginsenosides have exhibited promising potential in cancer therapy, the clinical application of the orally administered ginsenosides is limited by their poor bioavailability (<5%), given their poor water solubility, low oral absorption rate, and rapid blood clearance (Kim et al., 2018; Pan et al., 2018). Advances in nanotechnology and materials science have facilitated the development of nano-delivery systems for efficient and safe treatment of cancer and inflammatory diseases (van der Meel et al., 2019; Dawulieti et al., 2020; Shao et al., 2020; Hu et al., 2021; Wang H et al., 2021; Zhao et al., 2021; Shi et al., 2022; Tu et al., 2022). Ginsenoside nano-delivery systems have received considerable attention owing to their improved bioavailability, synergism, and detoxification (Shi et al., 2022; Tu et al., 2022). Several complex nano-delivery systems, including polymers, liposomes, metallic and inorganic particles, protein-based nanocarriers, and biomimetic nanoparticles (NPs), have been developed to improve the efficiency and safety of ginsenoside-based cancer therapy (van der Meel et al., 2019; Wahba et al., 2015; Hu et al., 2021; Wang H et al., 2021). However, most reported nano-delivery systems reportedly exhibit limited loading capacity, with additional concerns regarding their possible toxicity and biodegradation (Hong et al., 2020; Ren et al., 2020; Zhao et al., 2021; Mei et al., 2022; Xu et al., 2022).

Recently, carrier-free drug delivery systems, fabricated by self-assembly or co-assembly of pure drug molecules, have attracted substantial attention (Qin et al., 2017; Mei et al., 2022). Pure drug molecules without any chemical modification can spontaneously form uniform NPs by employing several methods (Zheng X et al., 2018; Sun et al., 2019; Zuo et al., 2021). Compared with free drugs, carrier-free nanodrugs afford improved bioavailability, prolonged blood circulation times, and superior tumor accumulation (Zheng et al., 2021). Compared with traditional nano-delivery systems, carrier-free drug delivery systems exhibit ultra-high drug loading (more than 60% or even 100%) and superior safety profiles (Huang et al., 2021). In addition, the co-delivery of dual or multiple drugs in a carrier-free form can help overcome the most intractable challenges encountered during cancer treatment (Wang M. Z et al., 2021).

In addition to their bioactive nature, ginsenosides have a potential role in stabilizing the phospholipid bilayer owing to their unique structure, comprising hydrophobic sterols and hydrophilic glycosides. The amphiphilic properties of ginsenosides can be regulated by the number of glycosides that may assemble with each other to form NPs. Based on these findings, we fabricated ginsenoside-based nanodrugs using a carrier-free method. We optimized the combination of ginsenosides Rg3 and Rb1 using self-assembly technology. The physicochemical and drug release properties of Rg3-Rb1 NPs were determined, and their therapeutic effects against TNBC were evaluated *in vitro* and *in vivo* and further compared with free ginsenosides and an Rg3/Rb1 mixture to demonstrate the advantages of carrier-free nanodrugs. Our results suggested that Rg3-Rb1 NPs can enhance antitumor efficacy for treating TNBC.

## Materials and Methods

### Preparation and Characterization of Rg3-Rb1 NPs

Rg3-Rb1 NPs were prepared using a precipitation method. First, ginsenoside Rg3 was dissolved in dimethyl sulfoxide (DMSO). The final concentration of Rg3 in the DMSO solution was 5 mg/ml. Next, 1 ml of this solution and 5–25 mg of ginsenoside Rb1 powder (weight ratios of 1:1, 3:1, and 5:1, Rb1-Rg3, respectively) were mixed and stirred for 5 min at 600 rpm. Subsequently, 20 ml of deionized water was added to the mixture and stirred for 30 min at 900 rpm. The turbid mixture was dialyzed against ultrapure water for 48 h (molecular weight = 1,000) for purification. Finally, Rg3-Rb1 NPs were collected by lyophilization. The weight ratio of Rb1 to Rg3 was quantified as x:1 by high-pressure liquid chromatography (HPLC).

### Drug Release

The drug release behavior of Rg3-Rb1 NPs was evaluated, as described below. Briefly, 10 mg of Rg3-Rb1 NPs (weight ratios of 1:1, 3:1, and 5:1, Rb1-Rg3, respectively) was added to a dialysis bag (1,000 Da). Subsequently, the bag was placed in a conical flask containing 100 ml of phosphate-buffered saline solution (PBS, pH: 7.4 or 5.5). The flask was sealed with a clean rubber stopper and placed on a shaking table in a 37°C incubator for 48 h. The dialysate was collected at 0, 1, 3, 6, 12, 24, and 48 h and analyzed by HPLC to determine the amount of Rb1 and Rg3.

### Cytotoxicity Assay

Cells were first seeded into 96-well culture plates at a density of 5,000 cells/well and cultured overnight for full attachment to determine the cytotoxicity of Rg3-Rb1 NPs. Then, cells were treated with free Rb1, free Rg3, Rb1/Rg3 mixture, or Rg3-Rb1 NPs at different final concentrations, as displayed in Figures 3A–C, respectively. Both Rb1 and Rg3 were dissolved in the cell culture medium at a high final concentration (100 μg/ml). After treatment for 48 h, cell viability was assessed using the standard SRB method. The absorbance of saline-treated cells was set at 100%. The IC50 value of each drug was calculated using GraphPad Prism software.

### *In Vivo* Antitumor and Safety Experiments

All mice were treated in compliance with the Guide for the Care and Use of Laboratory Animals, and all procedures were approved by the Animal Care and Use Committee of Jilin University (China). Female BALB/c mice (8-week-old) were used to evaluate the *in vivo* pharmacological effects induced by Rg3-Rb1 NPs. First, equivalent volumes of 4T1 cells and Matrigel were mixed at 4°C to obtain cell suspension (10^6^ cells/ml). Then, 100 μL of the suspension was injected into the second mammary fat pad of mice. Three days later, mice were randomly divided into five groups and treated with saline, Rb1 (5 mg/kg, intravenously), Rg3 (1 mg/kg, intravenously), Rb1/Rg3 mixture (5 mg/kg of Rb1 and 1 mg/kg of Rg3, intravenously), or Rg3-Rb1 NPs (1 mg/kg, based on the concentration of Rg3, intravenously). Drug treatment and measurements of tumor size and body weight were performed every 3 days. Tumor volume was calculated using the following formula: V_tumor_ = 0.5 × (long diameter) × (short diameter)^2^.

All mice were sacrificed on day 22. Major organs, including the heart, kidneys, liver, lungs, and spleen, were collected and fixed for further hematoxylin and eosin (H&E) staining, and the number of metastatic lung nodules was recorded before fixation. After H&E staining, tissue sections were photographed. The obtained images, along with body weight curves and serum biochemistry indexes, such as alanine aminotransferase (ALT), aspartate aminotransferase (AST), blood urea nitrogen (BUN), and serum creatinine (CRE), were used to evaluate the biosafety profiles of Rg3-Rb1 NPs.

## Results and Discussion

**FIGURE 1:**
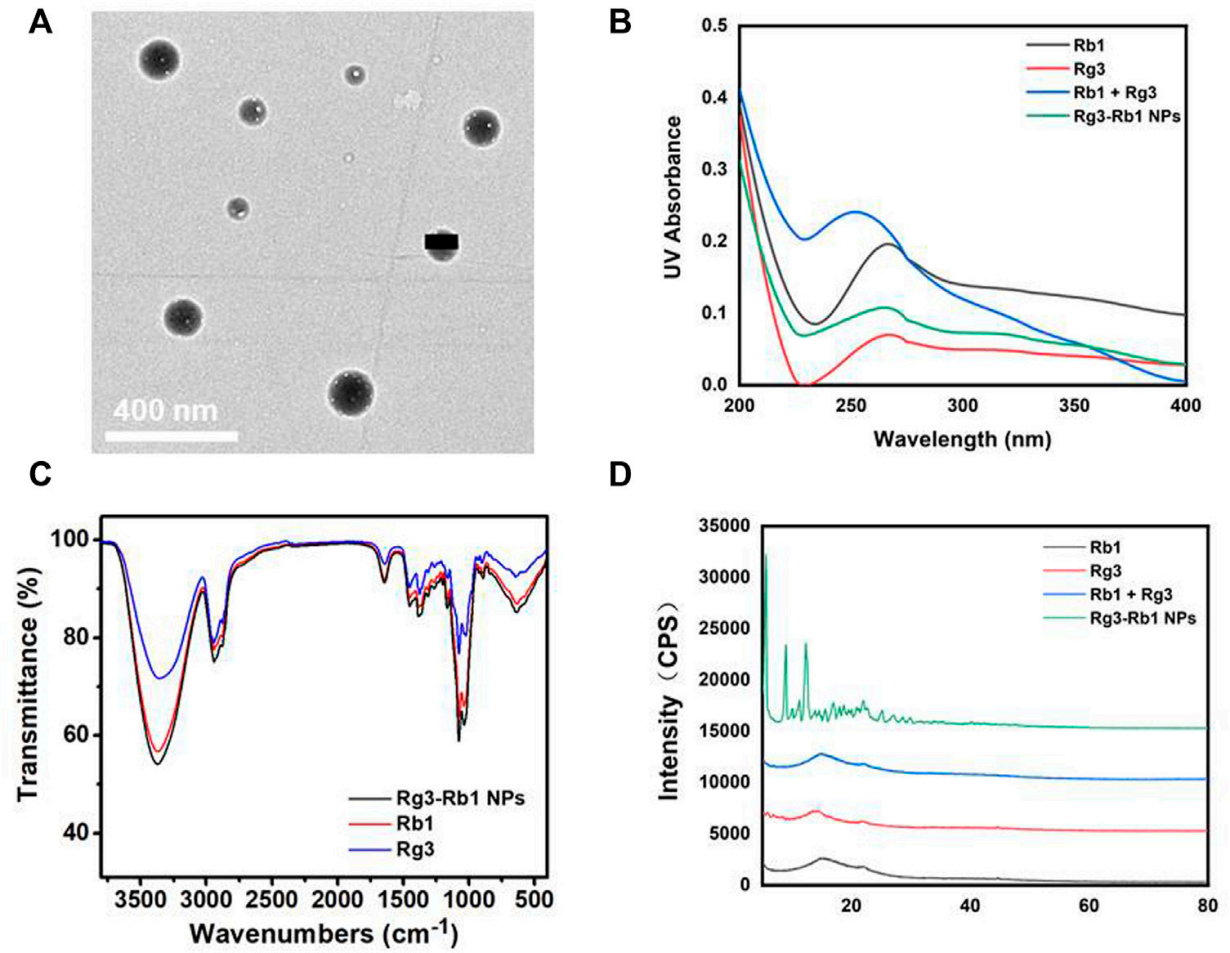
Characterizations of Rg3-Rb1 NPs. **(A)** TEM image of Rg3-Rb1 NPs. Scale bar = 400 nm. **(B)** UV-vis of Rb1, Rg3, Rb1+Rg3, and Rg3-Rb1 NPs. **(C)** FTIR of Rb1, Rg3, and Rg3-Rb1 NPs. **(D)** XRD spectra of Rb1, Rg3, Rb1+Rg3, and Rg3-Rb1 NPs. FTIR, Fourier-transform infrared spectroscopy; NPs, nanoparticles; TEM, transmission electron microscopy; UV, ultraviolet; XRD, X-ray diffraction.

To fabricate Rg3-Rb1 NPs, ginsenoside Rg3-Rb1 was mixed in DMSO at different weight ratios, and the mixture was added to water under constant shaking. Transmission electron microscopy (TEM) (Figure 1A) revealed that Rg3-Rb1 NPs exhibited a uniform nanosized morphology (diameter: 120 ± 20 nm), indicating the successful formulation of ginsenoside nanodrugs without chemical modifications or additional carriers. To explore the assembly mechanism, the ultraviolet- (UV-) vis spectra of Rg3-Rb1 NPs were recorded and compared with those of free Rg3 and Rb1 (Figure 1B). Raw Rg3 and Rb1 molecules exhibited similar absorption peaks at approximately 260 nm. In contrast, Rg3-Rb1 NPs displayed a wider and red-shifted absorbance peak, indicating a robust molecular interaction during the self-assembly process. Infrared spectral analysis showed that Rg3-Rb1 NPs possessed identical groups of Rg3 and Rb1 molecules (Figure 1C). Additionally, the X-ray diffraction pattern of raw Rg3 and Rb1 molecules showed a wide diffraction peak, indicating an amorphous crystalline structure (Figure 1D). Notably, Rg3-Rb1 NPs showed characteristic high-intensity diffraction peaks at 2θ values, ranging from 5° to 20°, indicating their naturally crystalline form. This phenomenon might be explained by the crystal orientations after recrystallization, which could be solubilized more easily, enhancing the dissolution rates when compared with their respective crystalline forms. Based on these results, we concluded that the formulated Rg3-Rb1 NPs were uniform nanodrugs containing Rg3 and Rb1.

**FIGURE 2:**
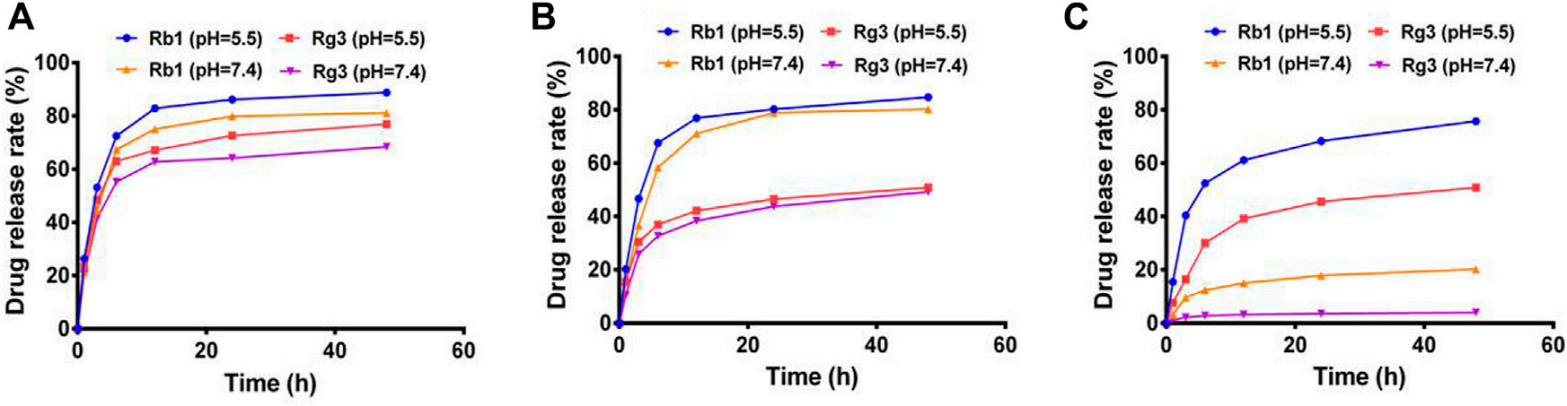
Drug release profiles of Rg3-Rb1 NPs of different weight ratios (Rb1 to Rg3) under acidic and neutral pH conditions. **(A)** 1:1, **(B)** 3:1, and **(C)** 5:1. NPs, nanoparticles.

To optimize the preparation of Rg3-Rb1 NPs, we investigated the self-assembly of Rg3 and Rb1 at different weight ratios. The release profiles of the three types of Rg3-Rb1 NPs were evaluated at a pH value of 7.4 to mimic normal conditions in bodily fluids or a pH value of 5.5 to mimic the acidic tumor microenvironment. As shown in Figures 2A–C, Rg3 release showed a time-dependent sustainable behavior, with a slight initial burst (more than 20%, at 0–6 h) in a buffer of pH 7.4. This initial burst in the Rg3 release might be attributed to establishing a balance in equilibrium between the inside and outside release settings. However, on decreasing the pH to 5.5, Rg3 release increased substantially, reaching 50.9% over a 48 h period. The increased drug release rate at low pH may be attributed to the facilitated dissociation of ginsenosides. Collectively, Rg3-Rb1 NPs controlled drug release, which might improve the bioavailability and therapeutic outcomes of ginsenosides. Based on the sustained-drug release behavior in the simulated acidic tumor microenvironment, the optimal self-assembly of Rg3-Rb1 NPs was prepared and used for subsequent experimental treatments at a mass ratio of 5:1 (Rb1:Rg3).

**FIGURE 3:**
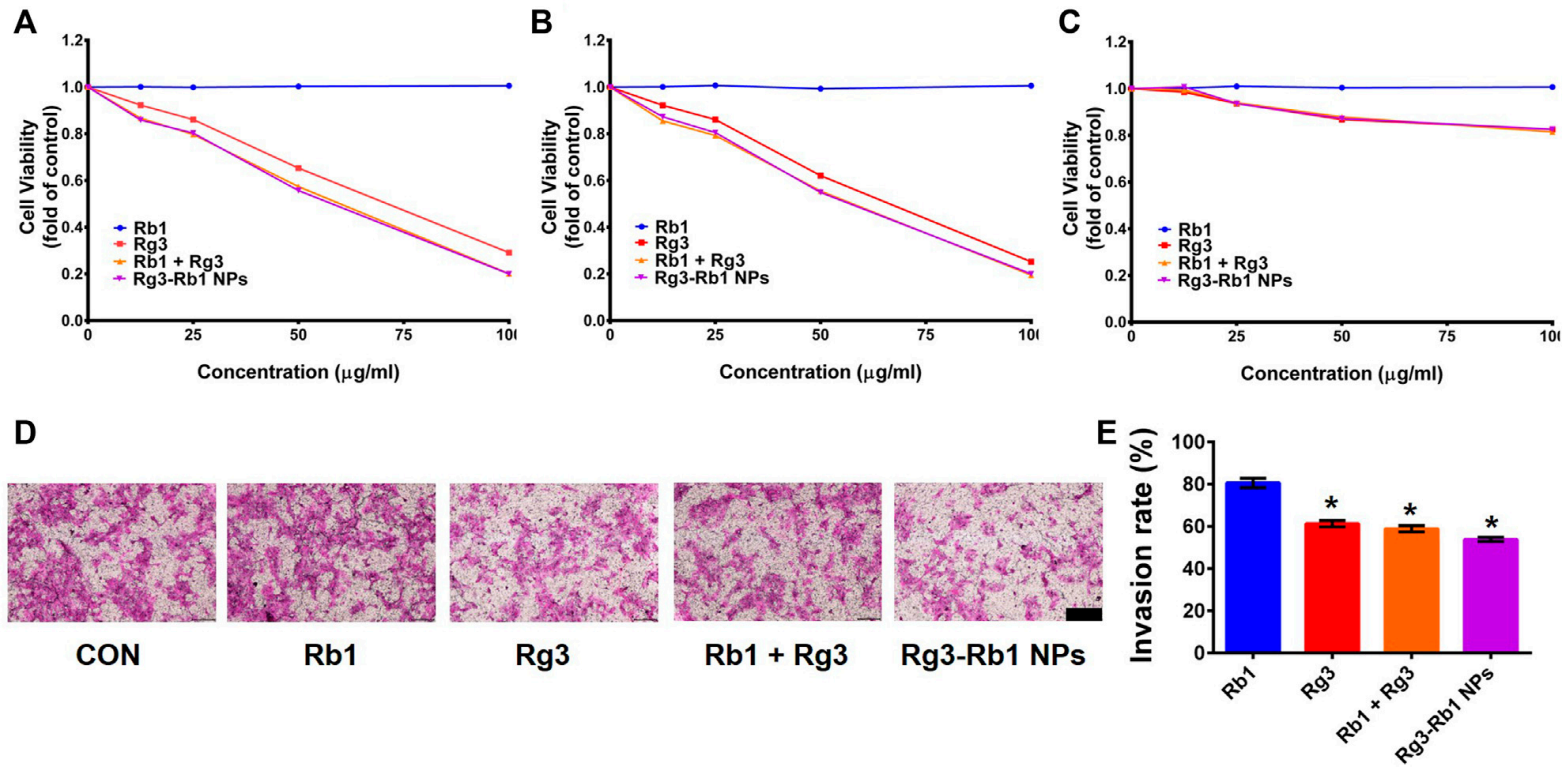
*In vitro* pharmacological effect of Rg3-Rb1 NPs. Cell viability of **(A)** 4T1, **(B)** MDA-MB-231, and **(C)** MCF-10a cells after 48 h of treatment. **(D)** Representative images and **(E)** quantitative analysis of crystal purple-stained invasive 4T1 cells. Scale bar = 200 μm ∗*p* < 0.05 compared with the control group. NPs, nanoparticles.

Next, we used the SRB assay to determine the viability of the mouse breast cancer cell line (4T1), human TNBC cell line (MDA-MB-231), and non-neoplastic breast cell line (MCF-10A) after 24 h of treatment with various concentrations of free Rg3, free Rb1, Rg3/Rb1 mixture, and Rg3-Rb1 NPs. Compared with the control group, the cell viability of all Rg3-containing groups dose-dependently decreased in 4T1 and MDA-MB-231 cells (Figures 3A,B), whereas free Rb1 induced minimal toxicity on breast cancer cells when compared with Rg3. The IC_50_ values in 4T1 cells were 63.87 ± 4.55 μg/ml for free Rg3, 60.14 ± 4.89 μg/ml for Rg3/Rb1 mixture, and 58.17 ± 4.14 μg/ml for Rg3-Rb1 NPs. Similarly, the IC_50_ values in MDA-MB-231 cells were greater than 100 μg/ml for the Rg3/Rb1 mix and Rg3-Rb1 NPs. These results indicated that combining Rg3 and Rb1 afforded a significantly greater decrease in toxicity than that induced by free ginsenosides, partly due to Rb1-mediated sensitization to chemotherapy. However, there were few differences between the Rg3-Rb1 NPs and Rg3+Rb1, which might be attributed to the prolonged incubation time and conditions *in vitro*. It is also worth noting that Rg3-Rb1 NPs, as well as the Rg3+Rb1 mixture, induced fewer toxic effects on normal TNBCs cells than MCF-10A cells (Figure 3C). The cell-protective effects of ginsenosides could explain this phenomenon. Taken together, these results indicated that the Rg3-Rb1 NPs provided a greater killing effect on TNBCs, with markedly diminished toxicity on normal breast cells. As shown in Figures 3D,E, Rb1-mediated sensitization to Rg3 inhibited 4T1 cell invasion (80.6%, 61.32%, 58.84%, and 53.79% for Rb1, Rg3, and Rg3/Rb1 mixture and Rg3-Rb1 NPs, respectively). In particular, Rg3-Rb1 NPs showed more efficient inhibition of invasion than Rg3+Rb1.

**FIGURE 4:**
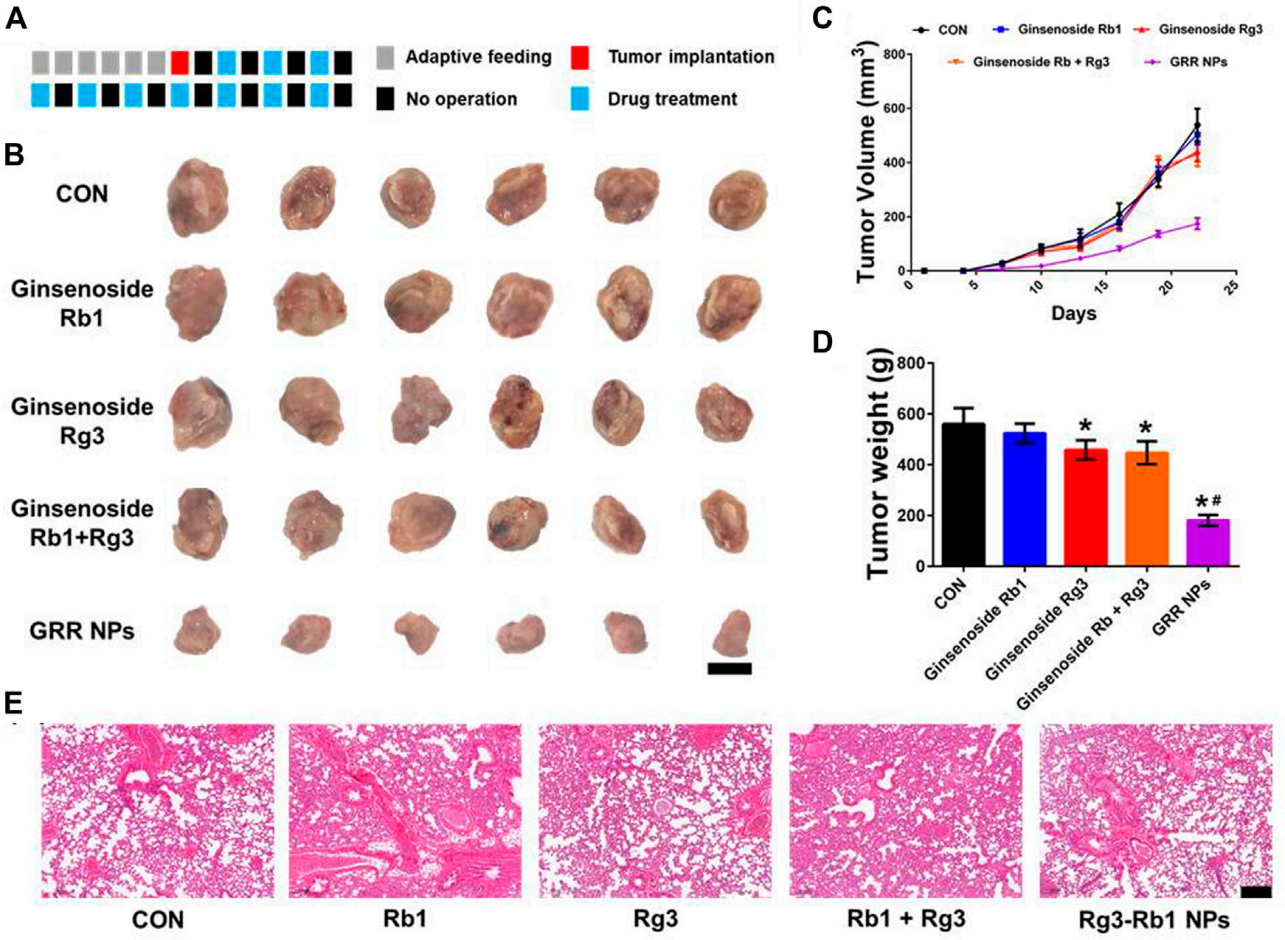
*In vivo* pharmacological effect of Rg3-Rb1 NPs. **(A)** The dosage regimen for treating the 4T1 orthotopic mammary mouse model, each square represents 1 day, **(B)** tumor images, **(C)** tumor volume, and **(D)** tumor weight. Scale bar = 1 cm. ∗*p* < 0.05 compared with the control group. #*p* < 0.05 compared with the Rg3 group. **(E)** Lung images of 4T1 orthotopic mammary mouse. Scale bar = 2 mm. NPs, nanoparticles.

Based on these findings, we examined the therapeutic efficacy of Rg3-Rb1 NP-mediated TNBCs *in vivo*. Herein, 4T1 orthotopic mammary tumor-bearing mice were intravenously administered saline (control group), free Rg3, free Rb1, Rg3+Rb1 mix, and Rg3-Rb1 NPs (Figure 4A). Compared with the control group, we observed that free Rg3, free Rb1, Rg3/Rb1 mixture, and Rg3-Rb1 NPs decreased tumor growth and reduced tumor weights at the end of the treatment period (Figures 4B–D); however, free Rg3 and Rb1 did not significantly impact tumor growth. Rg3-Rb1 NPs exhibited the strongest tumor growth inhibition and the lowest number of metastatic lung nodules (Figure 4E; Supplementary Figure S1). Notably, Rg3-Rb1 NPs demonstrated superior therapeutic efficacy to the Rg3/Rb1 mixture, indicating the advantages of self-assembled nanodrugs. Consistently, *in vivo* findings revealed that Rg3-Rb1 NPs exhibit markedly robust efficacy against TNBC.

**FIGURE 5:**
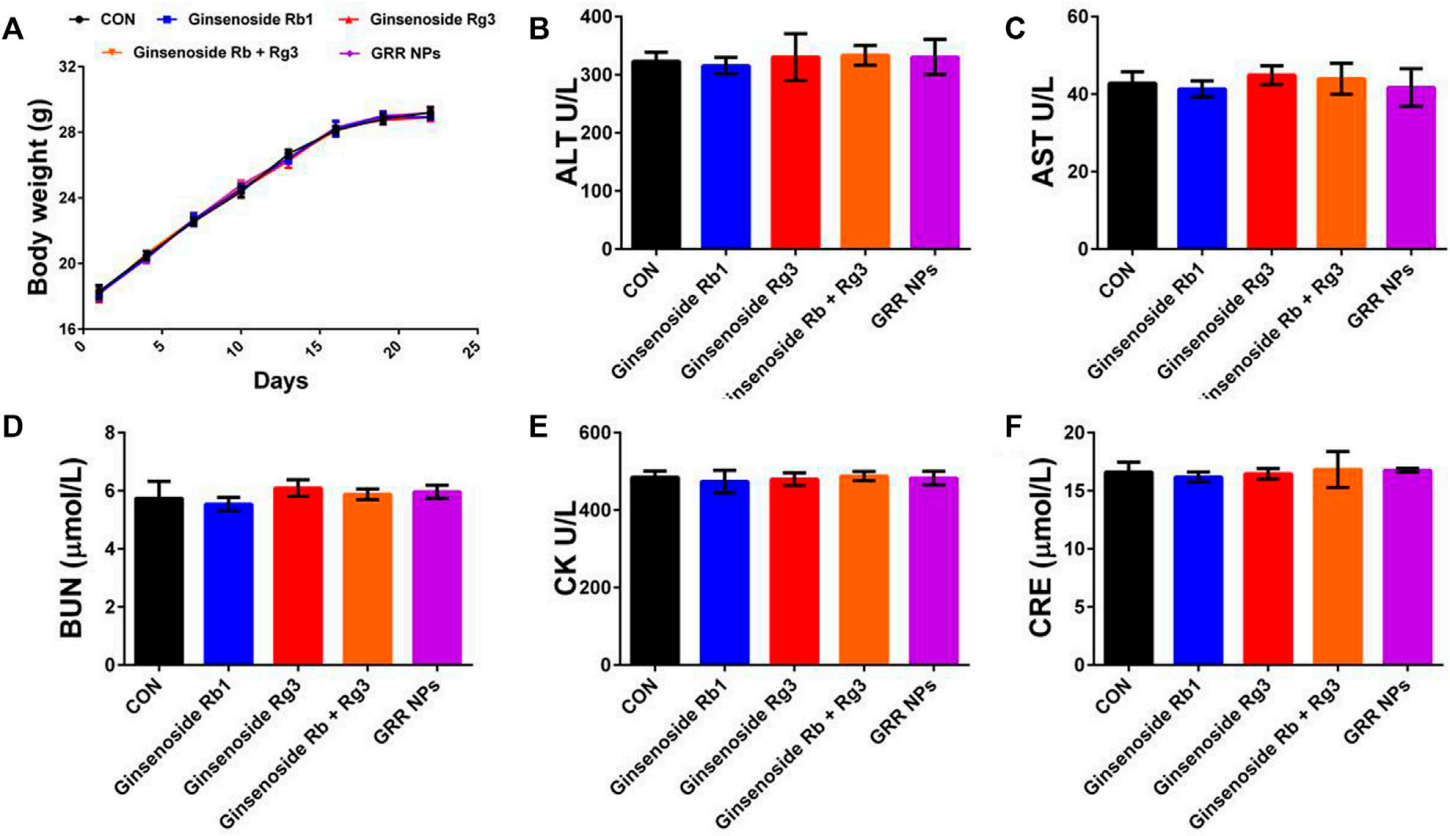
Biosafety effect of Rg3-Rb1 NPs. **(A)** Body weight, **(B)** ALT, **(C)** AST, **(D)** BUN, **(E)** CK, and **(F)** CRE of 4T1 orthotopic mammary mouse. ALT, alanine aminotransferase; AST, aspartate aminotransferase; BUN, blood urea nitrogen; CRE, serum creatinine; NPs, nanoparticles.

**FIGURE 6:**
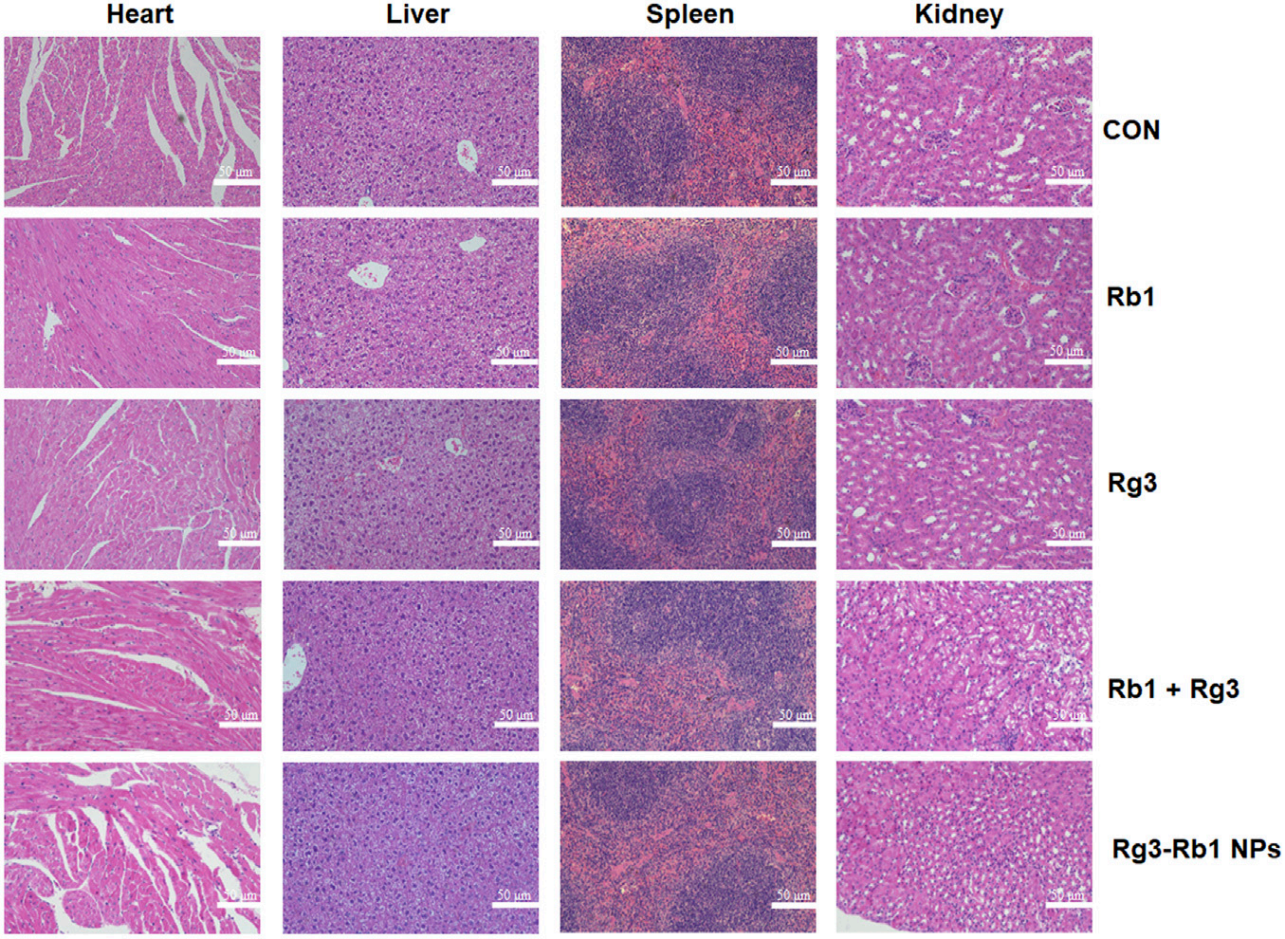
Hematoxylin and eosin-stained image of 4T1 orthotopic mammary mouse. Scale bar = 50 μm.

Finally, we performed a toxicological evaluation to determine whether Rg3-Rb1 NPs generate toxic side effects in the body following oral administration. The weight-change curve revealed that no treated group exhibited a significant effect (Figure 5A). Based on serum biochemistry assays, including assays for ALT, AST, BUN, and CRE, we detected no significant differences between the ginsenoside-containing and control groups (Figures 5B–F). In addition, no pathological damage was observed in primary organs, including the liver, spleen, kidney, and heart, across all groups (Figure 6). Overall, these safety profiles suggested that Rg3-Rb1 NPs exhibit low systemic toxicity.

In summary, we developed carrier-free ginsenoside nanodrugs composed of Rg3 and Rb1 using a simple nanoprecipitation method. Rg3-Rb1 NPs demonstrated a greater antitumor and anti-invasive effect against TNBC cells (vs. free ginsenosides), along with less toxicity in normal breast cells. The *in vivo* experiments clearly revealed that Rg3-Rb1 NPs preferentially inhibited 4T1 tumor growth and lung metastasis with a good biosafety profile. Overall, the Rg3-Rb1 NPs might have superior antitumor and antimetastatic properties, which might provide insight into the development of ginsenoside nanodrugs for safe and effective TNBC management.

## Data Availability

The raw data supporting the conclusion of this article will be made available by the authors, without undue reservation.
