# Supplementary material for: Fabrication of Ginsenoside-Based Nanodrugs for Enhanced Antitumor Efficacy on Triple-Negative Breast Cancer
Source: Front Bioeng Biotechnol. 2022 Aug 12;10:945472. doi: 10.3389/fbioe.2022.945472 (PMC9412961; doi:10.3389/fbioe.2022.945472)
Supplement: Supplementary file 1 [file DataSheet1.docx]

Supplementary Material

Fabrication of Ginsenoside-based Nanodrugs for Enhanced Anti-tumor Efficacy on Triple Negative Breast Cancer

**Shuting Zuo^1^, Zhengyu Wang^1^, Xianquan An^2^, Jing Wang^1^, Xiao Zheng^3*^, and Yan Zhang^1, *^**

^1^ Department of Breast Surgery, The Second Hospital of Jilin University, Changchun,130041, China;

^2^ Department of anesthesiology, The Second Hospital of Jilin University, Changchun,130041, China;

^3^ School of Biomedical Sciences and Engineering, South China University of Technology, Guangzhou 510006, China.

***Correspondence:**Prof. Yan Zhang, Department of Breast Surgery, The Second Hospital of Jilin University, Changchun,130041, China. E-mail addresses: zhangy01@jlu.edu.cn.

Dr. Xiao Zheng, School of Biomedical Sciences and Engineering, South China University of Technology, Guangzhou 510006. E-mail: zhengxiao129@scut.edu.cn.

## Chemicals and Reagents

Ginsenoside Rb1 and Rg3 (purity: >95%) were purchased from Chengdu. Sulforhodamine B (SRB) were obtained from Sigma-Aldrich (St. Louis, MO, USA). Dulbecco's Modified Eagle Medium (DMEM), fetal bovine serum (FBS), trypsin and penicillin-streptomycin (10,000 U/mL) were obtained from GIBCO (Carlsbad, CA, USA). Matrigel was purchased from Corning Inc. (Billerica, MA, USA). Assay kits for determing alanine aminotransferase (ALT), aspartate aminotransferase (AST), alkaline phosphatase (ALP), blood urea nitrogen (BUN) and creatinine (CRE) were obtained from Nanjing Jiancheng Bioengineering Institute (Nanjing, Jiangsu, China). All reagents were directly used without any further purification.

## Characterization of Rg3-Rb1 NPs

The morphology of Rg3-Rb1 NPs was imaged by a transmission electron microscope (JEOL, Ltd., Japan). UV–vis absorption spectra were obtained using a Shimadzu 3100 UV–vis spectrophotometer. Fourier transform infrared (FTIR) spectra were performed with a Nicolet AVATAR 360 FTIR instrument. X-ray powder diffraction (XRD) investigation was carried out on a Rigaku X-ray diffractometer using Cu Kα radiation. A Nano-ZS 90 Nanosizer (Malvern Instruments Ltd., Worcestershire, UK) was used to determine the size distribution and zeta potential of Rg3-Rb1 NPs.

**Cell Culture**

Mouse breast cancer cell line (4T1), human TNBC cell line (MDA-MB-231) and a non-neoplastic breast cell line (MCF-10A) were purchased from the American Type Culture Collection. All cells were cultured in DMEM with 10% FBS, 100 U/mL penicillin and 100 U/mL streptomycin in a humidified incubator with an atmosphere of 5% CO_2_.

## Invasion assay

Invasion assay were performed to determine the metastasis inhibition effect of Rg3-Rb1 NPs. Briefly, 4T1 cells were seeded into 24-well plates at the density of 20000 cells per well and cultured overnight for fully attaching. Then cells were treated with saline, 50 µg/mL of free Rb1, free Rg3, Rb1/Rg3 mixture or Rg3-Rb1 NPs, respectively. After 24 hours, culture medium was removed. Cells were washed with PBS for twice. Fresh culture medium with 5% FBS was added into each well. Next, 20 µL of Matrigel (0.5 mg/mL) dissolved with fresh culture medium was applied evenly on the top side of chamber and cultured for overnight in a 37℃ incubator, and then 5000 of 4T1 cells mixed with 1% FBS-contained culture medium were seeded into upper chamber (8.0 µm). Chambers were placed into the forementioned well. After co-cultured for 24 hours, cells attached onto the bottom side of the chamber were stained with crystal purple and observed under a microscope. Cells on the top side were then gently removed. Chambers were washed with 33% acetic acid (w/v), and optical density (OD) of eluent were measured by a microplate reader under the wavelength of 570 nm. Mean OD value of cells from both sides of control group was set as 100%. Invasion rate was calculated with following formula: OD_bottom_/(OD_con top_ + OD_con bottom_) × 100%.

## Statistical analysis

All experiments were performed at least three times and results were exhibited as mean ± standard deviation. Comparison between groups were calculated by Student’s t-test (two groups) or Bonferroni’s post hoc test (three groups or more). Data were analyzed on SPSS software. Differences were considered statistically significant when P-values were less than 0.05.


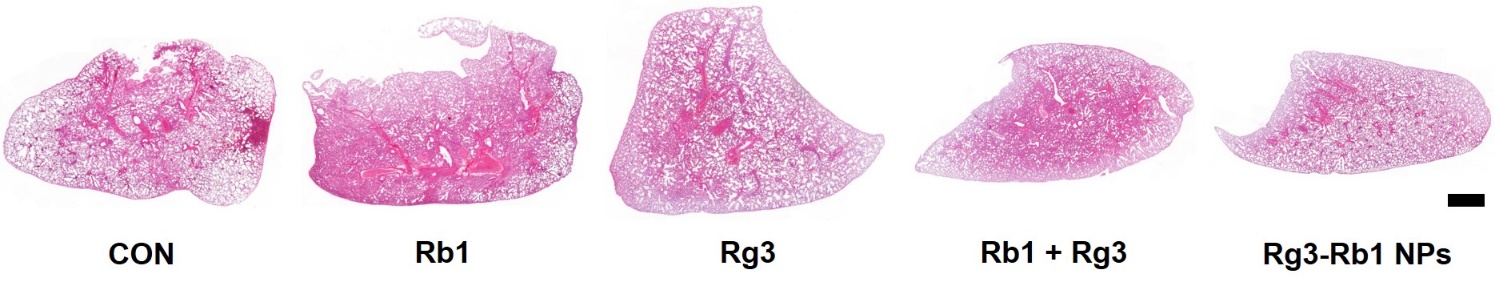


**Figure S1.** Hematoxylin and eosin-stained image of lungs. Scale bar=10 mm.
